# Unraveling Healthy Longevity: Characterizing Participants Aged 80+ in the Million Veteran Program

**DOI:** 10.1093/geroni/igaf122.4042

**Published:** 2025-12-31

**Authors:** Xuan-Mai Nguyen, Yanping Li, April Williams, Jane Driver, Kelly Cho, J Michael Gaziano, Stacey Whitbourne, Ariela Orkaby

**Affiliations:** VA Boston Healthcare System, Boston, Massachusetts, United States; VA Boston Healthcare System, Boston, Massachusetts, United States; VA Boston Healthcare System, Boston, Massachusetts, United States; VA Boston Healthcare System, Brockton, Massachusetts, United States; VA Boston Healthcare System, Boston, Massachusetts, United States; VA Boston Healthcare System, Boston, Massachusetts, United States; VA Boston Healthcare System, Boston, Massachusetts, United States; Brigham & Women’s Hospital, Harvard Medical School, Boston, Massachusetts, United States

## Abstract

US Veterans aged 80 and older represent one of the fastest growing segment of the aging population, however the functional status and lifestyle behaviors of this population is poorly understood. We extracted survey and health record data from participants in the Department of Veterans Affairs (VA) Million Veteran Program who were 80 years and older upon enrollment. Descriptive statistics were reported for demographics, morbidities and functional status (basic Activities of Daily Living (ADL), Instrumental Activities of Daily Living (IADL), gait speed, requiring the assistance of another person, and the VA Frailty Index (VA-FI)). Adherence to healthy lifestyle behaviors (healthy diet, adequate physical activity, social support, avoiding opioids, not smoking, restful sleep, no heavy drinking, good stress management) was also reported. There were 84,152 participants aged 80 and older. Median age was 84 years (range: 80-109; IQR: 81, 87), 98% were male and 96% were non-Hispanic White. Few (10%) required assistance for 1+ ADLs and 24% required assistance for 1+ IADLs. Almost half (47%) reported a gait speed of normal (2-2.9 mph) or faster, 9% required the assistance of another person, and 36% were classified as robust using the VA-FI. Participants reported adhering to a median of 5 (IQR 4, 6) healthy lifestyle behaviors. This MVP cohort is among the largest group of adults over age 80 and captures a highly functional group with good adherence to healthy lifestyle factors. Future work will better characterize modifiable risk factors and genetic information to guide healthy aging for all.

